# Analyzing the Effectiveness of the Brain–Computer Interface for Task Discerning Based on Machine Learning

**DOI:** 10.3390/s20082403

**Published:** 2020-04-23

**Authors:** Jakub Browarczyk, Adam Kurowski, Bozena Kostek

**Affiliations:** 1 Faculty of Electronics, Telecommunications and Informatics, Gdansk University of Technology, Narutowicza 11/12, 80-233 Gdansk, Poland; jakbrowa@student.pg.edu.pl; 2 Multimedia Systems Department, Faculty of Electronics, Telecommunications and Informatics, Gdansk University of Technology, Narutowicza 11/12, 80-233 Gdansk, Poland; adakurow@multimed.org; 3 Audio Acoustics Laboratory, Faculty of Electronics, Telecommunications and Informatics, Gdansk University of Technology, Narutowicza 11/12, 80-233 Gdansk, Poland

**Keywords:** electroencephalography (EEG), brain–computer interface (BCI), feature extraction, automatic classification, deep learning

## Abstract

The aim of the study is to compare electroencephalographic (EEG) signal feature extraction methods in the context of the effectiveness of the classification of brain activities. For classification, electroencephalographic signals were obtained using an EEG device from 17 subjects in three mental states (relaxation, excitation, and solving logical task). Blind source separation employing independent component analysis (ICA) was performed on obtained signals. Welch’s method, autoregressive modeling, and discrete wavelet transform were used for feature extraction. Principal component analysis (PCA) was performed in order to reduce the dimensionality of feature vectors. *k*-Nearest Neighbors (kNN), Support Vector Machines (SVM), and Neural Networks (NN) were employed for classification. Precision, recall, F1 score, as well as a discussion based on statistical analysis, were shown. The paper also contains code utilized in preprocessing and the main part of experiments.

## 1. Introduction

The spontaneous electrical activity of the brain acquired from electrodes placed on the human scalp in a noninvasive manner is extensively explored in many areas of interest, to name a few: neuroscience, cognitive science, emotion recognition, gaming experience, etc. [1,2]. Research on the brain–computer interface (BCI) was primarily motivated by supporting interaction with the environment of disabled people [3,4,5]. Moreover, examples such as detecting and classifying epileptic seizures based on EEG signals [6], controlling driver fatigue [7], sleep disturbance detection [8], recognizing different mental states [8,9], etc. are of great importance.

The practical implementation of the brain–computer interface (BCI) systems uses electroencephalographic (EEG) signals [7,10,11,12]. In BCI systems, the recorded signal is preconditioned in order to eliminate the artifacts and interferences, among others, resulting from eye blink, eye movement, muscle activity, or signal drift due to electrode misplacement [1,13,14,15,16]. Optionally, the signal can also be subjected to a blind source separation procedure. Such methods as Independent Component Analysis (ICA) are used for this purpose [17,18,19,20,21,22,23,24,25,26]. Then, extraction of features, i.e., reduction of the signal to a vector of parameters of lower dimensionality, is performed [27,28,29]. Such a reduction enables to distinguish signals representing different types of mental activity that the BCI system is to recognize [10,30]. However, in deep learning classification, feature extraction is not always applied as signal characteristics may be automatically derived from autoencoders [31,32]. Moreover, Wu et al. proposed an experimental scenario in which the feature selection and classification were performed simultaneously [33]. The method proposed was applied to the high-dimensional setting with the number of features larger than the number of samples [33]. Finally, machine learning methods, including both baseline algorithms such as k-Nearest Neighbors (k-NN), Random Forest [34], or Support Vector Machine (SVM) [35,36], as well as deep learning methods [37,38,39,40,41,42,43] are extensively employed in discerning mental state or classifying brain activity. Overall, it is evident that a hybrid approach is needed to classify the mental state regardless of the application area. Therefore, the most challenging issues related to recognizing mental states based on the recorded EEG signal are the selection of signal analysis and classification methods. In the most recent survey by Gu et al. [44], one may find references to BCI contributions to several fields of research and applications. A table containing an overview of EEG devices with their characteristics is given with adequate references. This survey presents a comparison between deep learning neural networks and traditional machine learning methods to prove the recent improvement of current deep learning algorithms in the EEG analysis. Overall, several topics are addressed by Gu et al., i.e., advances in sensors and sensing technologies, characteristics of signal enhancement and online processing, recent machine learning algorithms and the interpretable fuzzy models for BCI applications, state-of-the-art deep learning algorithms and combined approaches for BCI applications, and the evolution of healthcare systems and applications in BCIs [44]. Further, artifact removal techniques from the EEG signal are discussed along with the EEG signal analysis in real-time. Equally valuable, comprehensive, and thorough is a review prepared by Zhang et al. [35]. The focus of this survey is on advancement in applying deep learning to BCI as well as showing new frontiers. An important aspect of this review is to show details concerning EEG signal types under classification, along with the classification methods employed. Indeed, one should refer to this survey as it comprises a systematic review of brain signals and deep learning techniques for BCI. The paper discusses the popular deep learning techniques and state-of-the-art models for BCI signals, reviews the applications and remaining challenges of deep learning-based BCI, and finally, highlights some promising directions for future research. It is interesting to read also a survey source from 2010 [45], in which the impact of various events, namely, sleep, epilepsy, reflexology, drugs/anesthesia, diabetes, meditation, music, and artifacts, on the EEG signal is given. One of the most important topics contained in both surveys is related to transfer learning methodologies, which may be crucial in exploiting knowledge acquired to enhance the classification performance [35,44].

The survey by Zhang et al. examines 232 literature sources [35], and Gu et al. [44] provides 209 references; Google search returns a plethora of publications related to EEG-based BCI, thus it is not possible to follow all the threads presented. However, an attempt to recalling some works from the literature is made herein to include some selected sources to show that there does not exist one way of dealing with the EEG signals in terms of preprocessing, feature extraction (if any strategy applied), classification scheme, etc. On the basis of such a recollection, one may easily see the limitations of their own study and treat it as a starting point for future research directions.

Examples of the EEG-based classification performance obtained for various application tasks are given in Table 1, including the literature resources recalled in the survey by Zhang et al. [35] and Gu et al. [44] as well as some retrieved from other publications. 

For each study carried out, we have chosen in part, a classical approach to classification of the EEG signals (i.e., feature extraction/learning algorithm), and a deep learning model. To compare both approaches, the EEG signals acquired at our laboratory were utilized. We are aware that there exists a great number of datasets available to the public, examples of which are included in [49,51,53,61,66,67,68,69,70,71,72,73,74,75,76,77,78], and they could be employed, e.g., as test data or in transfer learning applied to deep learning. However, many of the cited works are also exploratory in their character [7,9,47,55,63], they include a variety of datasets, signal acquisition methods, data formats, etc., which cannot be directly compared to the outcome of the study performed by us. Therefore, we have decided to acquire our own locally acquired data, especially as the experiments also served other purposes.

The aim of the study presented is to create a practical framework for the automatic classification of mental states. It comprises both signal analysis and several selected classification algorithms. The classification schemes are compared as to their overall effectiveness of the automatic classification of mental states. For this purpose, EEG signals from 17 people in three different mental states—relaxation (called meditation), excitation (called music video), and solving logical task (called logic game)—are collected using an Emotiv EPOC+ helmet [79]. These raw signals were acquired from a set of standard positions: AF3, F7, F3, FC5, T7, P7, O1, O2, P8, T8, FC6, F4, F8, and AF4, according to the 10–20 (10%) extended electrode configuration on the scalp [80,81,82]. The acquired signals are separated by means of independent component analysis (ICA). For the extraction of features from the signals, the Welch method (for estimation of power spectral density (PSD) of a given time sequence), autoregressive modeling (Burg algorithm), and discrete wavelet transform (DWT) are selected. Such an approach is seen in many other literature sources [35,44,45,83]. The obtained feature vectors are reduced by Principal Component Analysis (PCA). For completing the EEG signal processing framework for classifying mental states, three classification methods are used: *k*-Nearest Neighbors (*k*-NN), Support Vector Machine (SVM), and Neural Network (NN), belonging to the category of deep learning. As pointed out in the survey of Zhang et al. [35], the recent advances in frontiers of deep learning-based BCI refer mostly to deep learning techniques, which is why in the classifiers employed in the carried out study, an NN was also included. However, it should be noted that this a simple model with three hidden layers and the LeakyReLU activation function is adapted in our study. 

The organization of this work is as follows. The following Section describes the dataset building and preprocessing to which the signals are subjected. Section 3 contains a thorough presentation of experiments, which consists of the EEG-based signal classification. Details regarding the technique used to reduce the dimensionality of feature vectors, given classifier settings and results obtained, are discussed. For performance evaluation, two schemes are executed: In the first one, an 80/20% split of the dataset into training/test sets is produced for *k*-NN and SVM, and a 70% training set, 10% validation set, and 20% test set for the NN algorithm. Moreover, 10-fold cross-validation for a more reliable assessment of classification performance is carried out on the best and the worst outcomes of the first validation scheme. This allowed us to check that the model can be trained repetitively with a similar result regardless of the choice of examples for training [84]. For each classifier performance, precision, recall, and F1 score are shown. Moreover, statistical analysis is performed for the experiments, resulting in appropriate metrics as well as indicating whether the differences obtained for two validation schemes are statistically significant. The paper also contains observations on limitations of the investigation carried out and possible ways to overcome them, as well as conclusions resulting from the conducted research. The prepared code snippets are contained in Appendix A and an attached zip file.

## 2. Materials and Methods

EEG signals of 17 subjects participating in the experiment were acquired. In the first stage of the research, the participants were instructed to relax. In the second phase, subjects watched the music video. In the last stage, subjects played a game involving logical thinking. For a given subject, durations of all stages were equal but varied between subjects. An Emotiv EPOC+ device equipped with 14 measuring electrodes was used to acquire the signals [79]. The sampling frequency was set to 128 Hz.

**Figure 1:**
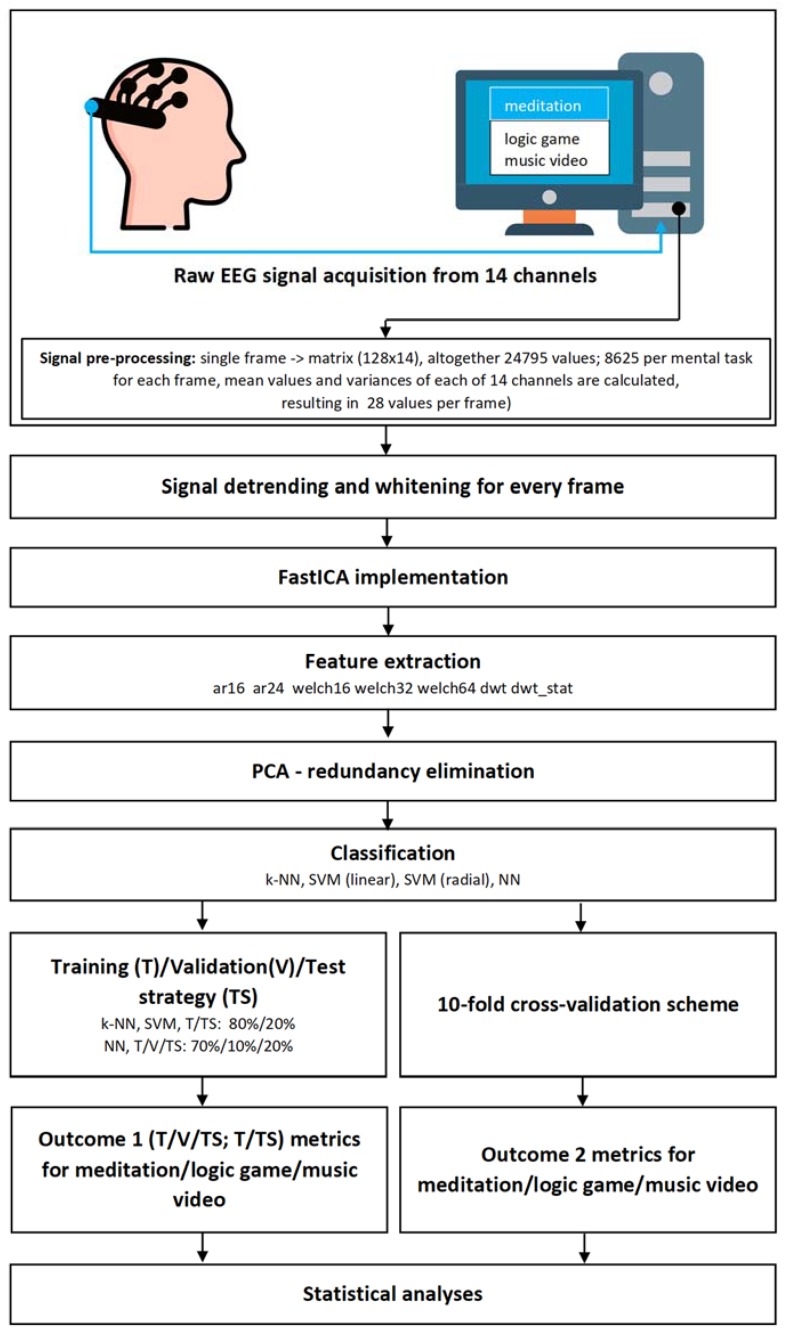
The flowchart of the study performed.

The article contains snippets of Python [85] code to illustrate performed operations. They are simplified versions of the code used for calculations. These snippets are contained in Appendix A; the code is also available to interested parties (see Supplementary Materials for the online address). The flowchart of the study performed is shown in Figure 1.

### 2.1. Building the Dataset

For each subject, the last 50 s of recorded signals, as well as 50 s of signals recorded between successive stages, were discarded. The remaining signals were divided into 1 s frames with a 0.5 s overlap. Thus, a single frame has the form of a matrix with dimensions (12,814). Each frame is assigned the corresponding category: meditation, music video, or logic game. The final number of frames was 24,795, i.e., 8265 for each category.

Overlap means that, for a given subject, the *l* last samples of *i*th frame of a given category from a given channel have the same values as the *l* first samples of *i* + 1 frame of that category and from that channel. The purpose of using overlap is dataset augmentation.

### 2.2. Data Preprocessing

For each frame, mean values and variances of each of 14 channels were calculated, giving 28 values per frame. They were saved for later use. Afterward, each channel of every frame was detrended using the *scipy.signal.detrend* function. Then, every frame was whitened and subjected to independent component analysis (ICA [17]) using the FastICA algorithm (see Appendix A).

Subsequently, for each channel in each frame, features were computed using feature extraction schemes described further on. Then, the feature vectors corresponding to subsequent channels were concatenated into one feature vector. Finally, previously computed mean values and variances were attached to the feature vector.

- ar16: for each channel of every frame, 16th order autoregressive models were computed using the Burg algorithm. The *arburg* function from the spectrum library was used for that. Only the real values of computed model coefficients were utilized (imaginary values were all equal to 0). After concatenating model coefficients from all channels with previously computed mean values and variances, final feature vectors of 252 elements were obtained. The code employed for the aforementioned calculations is contained in Appendix A.
- ar24: like ar16, but autoregressive models were of the 24th order. The final feature vectors contained 364 elements.
- welch16: for every channel in every frame, an estimate of power spectral density (PSD) was computed using the Welch method. Function welch from the *scipy* library was used for that. Samples from each channel in every frame were divided into eight nonoverlapping subframes of 16 samples each. Subsequently, nine coefficients were obtained per every channel. Final feature vectors (with pre-computed mean values and variances) contained 154 elements. Calculations were conducted with the use of the code shown in Appendix A.
- welch32: like welch16, but frames were divided into four nonoverlapping subframes of 32 samples each. Final feature vectors consisted of 266 elements.
- welch64: like welch16 and welch32, but frames were divided into two nonoverlapping subframes, each of 64 samples per channel. Final feature vectors consisted of 490 elements.
- dwt: each channel of every frame was decomposed using the 4th level discrete wavelet transform with db4 wavelet using the *wavedec* function from *pywt* library. The resulted vectors contained 14, 14, 22, 37, and 67 coefficients, respectively. After concatenating coefficient vectors with pre-computed mean values and variances, final feature vectors of 2184 elements were obtained. The transcription of this algorithm is provided in a listing contained in Appendix A.
- dwt_stat: each channel of every frame was decomposed with the discrete wavelet transform as in the dwt scheme. Subsequently, for each of five wavelet coefficient-based vectors, the following descriptive parameters are computed; mean value, mean value of absolute values, variance, skewness, kurtosis, zero-crossing rate, and the sum of squares (see Appendix A for the code used).

Dimensionalities of feature vectors obtained with the aforementioned schemes were reduced via principal component analysis (PCA). For each set of features derived from the training dataset, PCA was performed, retaining 95% of the variance in training data set features. Then, validation and test data were projected to PCA, which was written in Python (see Appendix A).

## 3. Experiments, Results, and Discussion

Experiments were carried out in order to compare the accuracy of test data classification using selected methods of feature extraction and classification. All computations were performed with the Python 3.5 programming language. The most important libraries used are *scikit-learn*, TensorFlow, and Keras [86,87,88].

First, the obtained dataset was randomly divided into training data, validation data, and test data in proportions of 70%, 10%, and 20%, respectively. The code snippet is shown in Appendix A. 

It should be noted that for each feature extraction scenario, two different schemes were computed. In the case of *k*-NN and SVM classifiers, the validation step was omitted, and validation data were used for training. Thus, PCA was performed on a total of 80% of available data for the k-NN and SVM classifiers, and 70% of available data for neural networks. After dimensionality reduction, the lengths of feature vectors for each scheme amounted to

- ar16: 38 in both cases,
- ar24: 61 in both cases,
- welch16: 61 in both cases,
- welch32: 110 in both cases,
- welch64: 204 in both cases,
- dwt: 1019 for *k*-NN and SVM, 1016 for neural networks, and
- dwt_stat: 136 in both cases.

Moreover, 10-fold cross-validation was executed to estimate further how the model is expected to perform on unseen data. These results are shown for comparison with the training data/validation/test scheme, but only for the best/worst feature extraction method/classifier variants.

### 3.1. Experiment 1: k-Nearest Neighbors

In the first experiment, *k*-NN classifiers were trained for chosen values of *k* using 80% of available data. The remaining 20% of data was used for testing. Accuracy was used as an effectiveness measure. Precision, recall, F1 score, and confusion matrices were used as auxiliary score measures. Code snippets for training classifiers, test data classification, and computing score measures are shown in Appendix A.

The results obtained in this experiment are presented as a summary in Table 2, and a discussion carried out through this Section. The best individual scores for the given feature extraction scheme and best mean score from all feature extraction schemes for a given *k* value are highlighted in bold.

In the conducted experiment, the highest classification accuracy of 63.86% was achieved for the welch32 scheme combined with the value of *k* = 11. Likewise, mean classification accuracy was also highest for the welch32 scheme. In general, schemes based on Welch’s method proved to be most effective. Although welch32 and welch64 schemes led to slightly better results than welch16, considering both average and individual scores, all three of them achieved the mean value of accuracy over 60%. Feature extraction schemes based on other used methods failed to get close to that score.

Autoregressive modeling-based schemes ar16 and ar24 achieved classification accuracy at the level of 50%. Interestingly, using the ar24 scheme resulted in slightly lower classification accuracy than using ar16. This shows that increasing the number of features may not provide higher accuracy.

Surprisingly, poor results were achieved using wavelet-based feature extraction schemes. The dwt scheme proved to be the least effective one in this experiment. Slightly better results, though still weak, were achieved with the dwt_stat scheme. A possible explanation for the poor performance of the dwt scheme may be overly high dimensionality of feature vectors, as dimensionality is thought to be particularly problematic in *k*-NN classifiers [89,90].

In the case of autoregressive modeling-based and wavelet transform-based feature extraction schemes, the best results were achieved with *k* = 17, the highest of used values. Welch method-based schemes were more effective with *k* = 11 and *k* = 14. It must be noted that the impact of the value of *k* on classification accuracy turned out to be small in comparison to the impact of the feature extraction scheme.

To find out the statistical significance of results presented in Table 2, a series of statistical tests was conducted. The approach employed for this purpose is a mixed linear model (MLM) [91]. Statistical testing with the use of MLMs allows testing of observations that are statistically dependent. In the case of data from Table 2, we test the difference of means obtained by the k-NN classifier with different types of feature extraction schemes. The averaging process is conducted over a set of values obtained for different values of *k*. The use of MLMs also allows testing of vectors of dependent values that analyze vectors of unequal length. This feature is important in the context of experiments 2 and 3, which have tables of results with missing values. For the calculation of MLMs, an implementation of this method provided in the Python *statsmodels* package [92] was employed. Columns from Table 2 were treated as dependent vectors of observations, thus the test describes the difference of performance of the *k*-NN algorithm for each type of input data preprocessing, and this difference is observed on a set of varied *k*-NN algorithm *k* hyperparameter values. The results of the test procedure are shown in Table 3. The algorithm finds the influence of each algorithm on the mean value of accuracy shown in the Table 2. The reference, which also defines values observed for the Intercept row from the table, is the welch32 algorithm, which was found to provide the highest mean accuracy calculated as a mean of performance for all variants of the *k*-NN algorithm.

Results of the analysis shown in Table 3 lead to the conclusion that all Welch-based classifiers had similar performance, and there are no statistically significant differences between them. This conclusion may be driven from both the value of z statistic and the associated *p*-value and from the confidence interval values, which are negative for the left boundary and positive for the right boundary. The significance level was assumed to be equal to the standard value of 0.05. The influence of the rest algorithms is negative, and the worst performance is found in the case of the dwt-based parameterization method, which, even in the most positive case of a value retrieved from the right boundary of the confidence interval, is worse than the left boundary of all other algorithms. Therefore, it can be concluded that the best performing group of parameterization is the one based on the Welch method, and there were no significant differences between algorithms from this group.

Below, a detailed discussion on examples of feature extraction schemes and classifier scenarios is shown. In Table 4 (left), a normalized confusion matrix for the 11-NN classifier and the welch32 feature extraction scheme is shown. Observations belonging to the meditation class were mostly correctly classified, while observations belonging to the music video and logic game classes were often confused with each other. Such a result is somewhat expected, as both watching the music video and solving logic puzzles involve a certain level of mental stimulation and require focusing the subject’s attention. Meditation, as the activity most different from the others, proved to be the easiest one to classify correctly. Confusion matrices for 11-NN welch16 and 11-NN welch64 (not presented in the article) contain very similar values. On the right side of Table 4 (right), a normalized confusion matrix for the 17-NN classifier and ar16 feature extraction scheme is shown. Again, the frames belonging to the meditation class are mostly correctly classified. Observations belonging to the logic game class are sometimes assigned to two remaining classes. Observations belonging to the music video class are least often correctly classified ones—only 32% of the observations of this class were correctly recognized. As many as 43% of the music video observations were misclassified as meditation. The confusion matrix for 17-NN ar24 (not shown in the article) contains very similar values.

In Table 5, normalized confusion matrices for 17-NN dwt and 17-NN dwt_stat scenarios are shown. These matrices differ greatly. In the case of 17-NN dwt, most observations of all classes have been recognized as logic game, a much lesser part as music video, and the least part as meditation. In the 17-NN dwt_stat scenario, the meditation observations were mostly correctly classified, while logic game and music video were assigned in different proportions to all classes, however most often to the meditation class. 

In Table 6, values of precision, recall, and F1 score for chosen scenarios are shown. Precision for a given class is defined as the ratio of the number of observations correctly assigned by a classifier to that class (true positives) to the number of all observations assigned by a classifier to that class (sum of true and false positives). Recall that a given class is defined as the ratio of true positives to the number of all observations belonging to that class (sum of true positives and false negatives). F1 score is defined in the following way.

(1) $$F1=2\cdot \frac{precision\cdot recall}{precision+recall}$$

In the case of data from Table 6, we also employed a series of statistical tests to find out the statistical significance of the obtained results. All confusion matrices used for calculation of precision, recall, and F1 score were also subject to the chi-square test, which is used to find if unevenness of value distribution in a given contingency table is uneven to a purely random chance or is it caused by some external factor. Confusion matrices in this context can be treated as a special case of contingency tables. For Table 6, only one result was found to be statistically insignificant and thus not recognized by the classification algorithm—a music video scenario in the case of the 17-NN dwt algorithm. The value of the test statistic was equal to 3.634, and thus the *p*-value is equal to 0.056. If the significance level of 0.05 is considered, the result of the classifier is equivalent to random assignment to the class, and the result is statistically insignificant. For the rest of the classifiers, the results are statistically significant. A Holm–Bonferroni correction for multiple testing was applied to the outcomes of the three consecutive tests conducted for each of the classes.

As earlier mentioned, results obtained with the use of the first validation scheme (training/validation/test or training/test) were compared to the outcomes of 10-fold cross-validation (2nd scheme). A confidence interval (α = 0.95) was calculated for a vector of values provided by the cross-validation procedure. Differences between the scores of both validation schemes are considered statistically significant if this value was outside the confidence interval. Calculations are performed with the use of R language. For calculation of confidence intervals, a DescTools library was employed [93]. 

If, in the 1st validation scheme, the result is outside the confidence interval, then the difference between this result and the nearer boundary of the confidence interval is taken into account. In our further discussion, if a performance measure value from the study based on the 1st scheme is below the lower boundary of the confidence interval, we will report an increased performance in the case of the cross-validation and provide the difference of performances according to the following formula,

(2) $${\Delta M}_{p}=CI_{L}-M_{3sets},$$

where ${\Delta M}_{p}$ is a difference between measures which can be accuracy, precision, recall, or F1; $CI_{L}$ is the value of the lower boundary of confidence interval calculated for results from cross-validation based assessment; and $M_{3sets}$ is the value of measure based on assessment employing single random division into training, validation, and test sets.

The formula is applied only if $CI_{L}>M_{3sets}$. If the degradation of performance is observed, then another formula is employed for reporting the result:

(3) $${\Delta M}_{p}=M_{3sets}-CI_{U},$$

where $CI_{U}$ denotes the upper boundary of confidence interval derived from outcomes of cross-validation based benchmark. This equation is applied only if $M_{3sets}>CI_{U}$.

#### Experiment 1

For comparison, 10-fold cross-validation was performed for the best (11-NN welch32) and the worst (17-NN dwt) training/test scheme in Experiment 1. The results are contained in Table 7. Comparing precision, recall, and F1 score metrics of the two test schemes, they are quite similar for 11-NN welch32. However, they differ for the logic game and music video for the 17-NN dwt. In the case of 10-fold cross-validation and 11-NN welch32, most of the observations belonging to the meditation class are classified correctly, while observations of remaining classes are assigned in nearly the same proportions to all classes. The statistical analysis performed for the comparison purpose between validation schemes is shown further on.

In the case of the worst scenario, when the DWT-based parametrization is considered, the accuracy value obtained in the 1st scheme was found to be within the confidence interval calculated from the results of cross-validation, thus there were no statistically significant differences between two approaches. Similarly, no such differences were found for the precision measure. However, for the recall measure, we found that the classifier performed significantly worse for the logic game class; the upper boundary of the confidence interval is 0.098 smaller than the result from the 1st scheme. However, the same classifier performed better in the case of the music video, and the improvement is very similar to the reduction of performance in the case of the logic game (i.e., 0.095). Obviously, a similar pattern can be observed for the F1 measure, which is derived from precision and recall. Performance for the logic game is statistically significantly worse (i.e., 0.018), and performance for music video increased by 0.048.

In the case of the best performing algorithm (based on the Welch method), we also did not find accuracy to be statistically different in both scenarios. Differences were observed for all remaining measures. For precision measure, an increase in performance was found for the logic game (i.e., 0.0158) and degradation for the music video class by 0.007. For the recall measure, performance degraded by 0.0445 for the logic game and increased by 0.0546 for the music video. For the F1 measure, performance for logic game degraded by 0.0052, and increased for meditation by 0.0028 and for music video by 0.0154. 

Observed changes of performances were statistically significant, but it is worth mentioning that in some cases, the difference between values from the 1st scheme and the closest boundary derived from the cross-validation assessment is small (smaller than 0.01).

### 3.2. Experiment 2: Support Vector Machines with a Linear Kernel

In the second experiment, the accuracy of classification with support vector machines was tested. A linear function was used as a kernel. Used values of penalty parameters C were 0.01, 0.1, 1, 10, and 100. For some combinations of C parameter value and feature extraction scheme, experiments were not conducted because of very long computation times, and poor results achieved for the given scheme in conjunction with other values of C. Data used for training and testing were the same as in Experiment 1. The code for training and testing classifiers is contained in Appendix A.

The results are shown in Table 8. The best individual scores for the given feature extraction scheme and best mean score from all feature extraction schemes for given *k* value are highlighted in bold. The highest value of accuracy was achieved for the welch32 feature extraction scheme, combined with the value of C = 1. It amounted to 66.71%, which is almost three percentage points higher than the best result in Experiment 1. The best mean value of the classification accuracy was achieved for the welch64 feature extraction scheme, although the score obtained with welch32 was not much worse. The highest mean scores of all feature extraction schemes were acquired for C = 10 and C = 100. This is probably because experiments were not conducted for wavelet-based feature extraction schemes, which would otherwise lower the mean scores.

Both the best individual and mean scores turned out to be slightly better than the scores obtained in Experiment 1. Nevertheless, similar conclusions can be drawn from both experiments. Welch’s method again turned out to be the best parametrization method in terms of both individual best and mean scores. The dwt scheme again turned out to be the least effective one. The main difference in the results of both experiments is that in Experiment 2, applying the ar24 scheme resulted in higher accuracy scores than using the ar16 scheme. The most substantial improvement in results was obtained for the dwt_stat scheme.

Similarly to the first experiment, an MLM-based analysis was also applied for data from Table 8. Results from such analysis are presented in Table 9. This table contains the results calculated with the mixed linear model analysis. In this case, a welch64 algorithm was employed as a reference.

Again, similar to the outcomes of the first experiment, Welch method-based algorithms performed similarly, and there were no statistically significant differences in their performance. The rest of the algorithms performed worse than the reference algorithm. The worst performance is associated with the dwt algorithm. 

Moreover, accuracies for the case of SVM (linear kernel) in 10-fold cross-validation were obtained for welch32 (the best performance) and dwt (the worst outcome) feature extraction variants. The results are shown in Table 10. Comparing these values with Table 8, one can observe that they are quite similar, though accuracy values are lower in the 10-fold cross-validation scheme. Again, the formal approach to statistical analysis will be shown at the end of this Section.

In Table 11 and Table 12, the normalized confusion matrices for welch32, ar16, dwt, and dwt_stat feature extraction schemes are shown. For the first three variants, confusion matrices are very similar to the ones obtained in Experiment 1. For the dwt_stat scheme, improvement in classification scores for observations belonging to logic game and music video in comparison to scores from Experiment 1 can be noted.

In Table 13, values of precision, recall, and F1 score for welch32, ar16, dwt, and dwt_stat schemes are presented. In all variants, with the exception of dwt values, all aforementioned measures are highest for the meditation class and lowest for the music video class.

Outcomes from Table 13 were also tested with the chi-square statistical test. Results from all variants were found to be statistically significant with the exception of dwt and C = 0.01. In this case, the statistic for meditation class was smaller than 0.001, which resulted in a *p*-value of 0.993; the logic game was associated with a test statistic of 0.629, which resulted in a *p*-value of 0.812; and the music video was associated with a test statistic value of 0.582 and a *p*-value of 0.812. Therefore, classification in this variant is equivalent to the class assignment done randomly, and outcomes are statistically insignificant.

In Table 14, precision, recall, and F1 score are shown for a 10-fold cross-validation scheme. Comparing these values with Table 13, one may observe that the metric values obtained for all classes look very similar; however, the statistical analysis shown below details whether the differences are statistically significant.

For the feature extraction method associated with the worst performance (based on DWT) we found that there were no statistically significant differences between the 1st scheme and the cross-validation based benchmarks. For the best performing scenario (based on welch32), we may observe that most of the results differ in a statistically significant way; however, some of the differences in performances are very small (smaller than 0.01). Overall accuracy was found to be lower for cross-validation (0.0012). Precision also provided smaller values for cross-validation (i.e., 0.0027). For recall, the performance also decreased for logic game and meditation (by 0.0206 and 0.0012, respectively). The performance for meditation increased by 0.0012. Degradation (0.0156) of the F1 score was observed for the logic game, and an increase of 0.003 in the F1 value was found for the meditation class.

### 3.3. Experiment 3: Support Vector Machines with Radial Basis Function Kernel

Experiment 2 was repeated using a radial basis function (RBF) as a kernel. The utilized values of RBF parameter γ were as follows, 0.1, 1, and 10. The code for training classifiers and test data classification is shown in Appendix A. A summary of results is presented in Table 15. The best individual scores and the best mean score for the given feature extraction scheme, C and γ are highlighted in bold.

The highest individual classification accuracy was again achieved for the welch32 scheme with parameters C = 10 and γ = 10. It amounted to 69.33%, a result that is over 2.5 percentage points better comparing to the linear SVM best score and 5.5 percentage points better than the *k*-NN best score. As in previous experiments, the scores obtained using the Welch method, in particular, in the welch32 and welch64 variants, turned out to be much higher than with the other methods. On the other hand, the mean accuracy scores of all C and γ values are not much higher for welch32 and welch64 variants in comparison to values obtained in Experiment 2. Moreover, for autoregressive modeling-based schemes and the dwt_stat variant, mean accuracy scores turned out to be much lower in comparison to scores obtained in previous experiments. This is due to the greater influence of C and γ on accuracy scores. In previously tested classifiers, changing the values of *k* and C parameters had a small impact on the accuracy of the classification. In the present experiment, the classification accuracy for the ar16 scheme with the parameters C = 1 and γ = 0.1 was 52.86%. After changing the value of γ to 10, classification accuracy amounted to only 33.33%. The difference is, therefore, almost 20 percentage points. As seen from Table 15, most of the used combinations of C and γ values resulted in relatively low classification accuracy compared to the maximum values, both in this experiment and in the previous ones, for a given feature extraction scheme. This explains the low average values of classification accuracy and indicates the need for fine-tuning of the SVM classifier parameters when using the RBF kernel.

In the performed experiment, the feature extraction scheme resulting in the poorest results turned out to be the dwt_stat scheme. The highest classification accuracy for this scheme decreased by 12 percentage points compared to the linear SVM classifier, and by six percentage points compared to the *k*-NN classifier. Using the radial basis function kernel resulted in a very different shape from the hyperplane decision boundary of the linear SVM classifier. The decision boundary of the *k*-NN classifier at high *k* values may converge to the hyperplane, which explains the similarity of the results for the *k*-NN and the SVM linear classifier. A different shape of achievable decision boundaries may result in better classification results in some data sets, but worse in others.

Results of the statistical MLM-based analysis of outcomes of the third experiment are presented in Table 16. In this table, the results of the mixed linear model analysis for data from Table 15 are contained. In this case, also the welch64 algorithm was treated as a reference.

Similar to the previous two experiments, no significant differences were observed for welch-based algorithms and the worst performance was found in the case of the dwt algorithm. However, the difference in performance between dwt and other algorithms such as ar16 and ar24 is not as prominent as in the case of previous experiments. In their case, pessimistic performance is similar to the pessimistic performance of the dwt algorithm.

10-fold cross-validation was also performed for SVM with a radial kernel function for two feature schemes based on the accuracy results (the lowest and the highest accuracies) contained in Table 17. Therefore, dwt_stat (C = 0.01, *γ* = 0.1) and welch32 (C = 10, *γ* = 10) cases were examined. Comparing Table 15 and Table 17, one can see that the results look very similar, though accuracy for the worst-performing algorithm (based on dwt stat) degraded by 0.0703.

In Table 18 and Table 19, the normalized confusion matrices for feature extraction schemes welch32, ar16, dwt, and dwt_stat with the best parameter combinations are shown. The confusion matrix for the welch32 scheme is very similar to the confusion matrix obtained for that scheme in previous experiments. The majority of meditation frames are correctly classified, while the other two categories are sometimes confused with each other.

The error matrix for the ar16 variant has similar values, as in the previous experiment. Classification accuracy for logic game and music video frames increased, while the accuracy for the meditation class decreased.

The confusion matrix for the dwt scheme, in turn, differs greatly from the matrices obtained in previous experiments, in which most of the observations were classified into the logic game or music video classes and very few observations into the meditation category. In the present experiment, most of the observations belonging to the meditation class are classified correctly, while observations of remaining classes are assigned in different proportions to all classes, but most often to the class meditation.

In the case of the dwt_stat scheme, observations belonging to the logic game and music video classes are assigned to three classes roughly equally. Observations of the meditation class are in half of the cases mistakenly assigned to other classes.

In Table 20, values of precision, recall, and F1 score for each signal class for the welch32, ar16, dwt, and dwt_stat feature extraction schemes are presented. For welch32 and ar16, the values of all measures are the highest for the meditation class and the lowest for the music video class. Note the relatively low precision for the meditation class and the dwt scheme.

After the statistical testing process with chi-square test, all differences presented in Table 20 were found to be statistically significant.

In Table 21, values of precision, recall, and F1 score are shown for 10-fold cross-validation for the best and worst results of the training/validation/test scheme, as shown in Table 20. For welch32, resulting metrics are very similar. For dwt_stat feature extraction scheme, values of all measures are lower. Again, the statistical analysis was performed showing which differences are statistically significant.

Degradation was found for all classes in terms of precision. The logic game deteriorated by 0.1526, music video by 0.1661, and music video by 0.2089. No differences were found for recall measure. In terms of the F1 measure, degradation was observed for meditation class (i.e., 0.0838) and music video (i.e., 0.1114).

Only two statistically significant differences were found for the best performing algorithm (based on Welch’s method). Both are associated with the recall measures. For the logic game, performance dropped by 0.0071, and there was an increase of 0.0033 for meditation. It is worth noting that these are low values compared to the magnitude of performance changes in other algorithms.

### 3.4. Experiment 4—Neural Networks

In the last experiment performed, the accuracy of classification using neural networks was examined. Neural networks belonging to deep learning classifiers class, with a single hidden layer with the ReLU activation function [94,95] and the softmax activation function in the output layer, were used. Weights were initialized with the He method (parameter kernel initializer = ’he uniform’) [96] was used, while biases were initialized with zeros. The Nesterov gradient method was used for training the network [97]. The learning rate parameter was set to 0.01 with a decay of 10^−6^ per epoch. Momentum was set to α = 0.9. To prevent overfitting, early stopping with the patience of 50 epochs was used. This parameter refers to the number of epochs to wait before early stop if no progress on the validation set is achieved. The maximum possible number of learning epochs was set to 2000. The results are presented in Table 22. The code used for training the networks is shown below.

For the autoregressive modeling-based and wavelet transform-based methods, the results obtained were similar to the results obtained with linear SVMs, while for Welch’s method, the obtained accuracy was even higher than in previous experiments. Again, the welch32 scheme, for which classification accuracy higher than 70% was achieved for the first time, turned out to be the best option. The code employed to achieve these outcomes is provided in Appendix A.

Values from Table 22 were also subject to statistical testing. To test it, the ANOVA analysis could be employed; however, first, a Levene test for uniformity of variance should be performed. The value of test statistic was equal to 0.883, and thus *p*-value was equal to 0.512. Therefore, all variances of observation vectors gathered for each algorithm can be assumed to be equal. Next, a series of Shapiro–Wilk tests were conducted to test the second assumption of the ANOVA test, which is Gaussian distribution of observation. For all but one algorithm *p*-value of the Wilk–Shapiro test was in a range between 0.157 and 0.629. However, for the dwt algorithm, the value of the Shapiro–Wilk algorithm was equal to 0.016, and therefore, it is concluded that one of the observations does not have Gaussian distribution, and the ANOVA test cannot be performed. *p*-values of the Shapiro–Wilk test were corrected for multiple testing with a Holm–Bonferroni correction. Instead of ANOVA, the Kruskal–Wallis nonparametric alternative for ANOVA has to be conducted. The statistic of the Kruskal–Wallis test is in this case equal to 67.454, and thus the *p*-value is smaller than 0.001, and differences between medians of results obtained by each algorithm are statistically significant in the case of at least one pair of algorithms. To find out such pairs, the Dunn post-hoc test is conducted. The matrix of *p*-values of the Dunn test is presented in Table 23.

Ar16 and ar24 performed similarly, and no statistical difference was found between the performance of those two algorithms. The behavior of the group of welch-based algorithms was close to ar-based schemes; however, a statistically significant difference was found between welch16 and welch32 algorithms. Although dwt and dwt_stat algorithms performed in a similar manner, no statistically significant differences in performance were found in their case.

Due to the fact that in each of the conducted experiments, the welch32 scheme provided the best results, further part of the experiments focused on tuning the neural network to obtain the best possible outcome with this scheme.

The general specification of the neural networks for which the best results were obtained is presented in Table 24. All described networks have output layers consisting of three neurons with the softmax activation function. In all networks, weights were initialized with the He method, while biases were initialized with zeros. The Nesterov gradient method was used to train the networks. The best result of all performed experiments is marked in bold. While using the 10-fold cross-validation scheme, the accuracy for the best neural network configuration resulted in a value of 0.7412. Thus, the outcome is very similar in both testing/validation schemes. 

In Table 25, the normalized error matrix for the best neural network is presented. It can be seen that better classification results compared to the SVM (RBF) classifier are due to the higher sensitivity for the music video category. Sensitivity for the other classes remained at a similar level. The left side of Table 25 shows results for the training/validation/test scheme, whether the outcomes of 10-fold cross-validation are contained on the right side. As seen in Table 25, the above conclusions are valid for both testing schemes.

In Table 26, values of precision, sensitivity, and F1 score for each class for the best neural network configuration are shown. Similarly, as in the previous experiments, scores are the highest for the meditation class and the lowest for the music video class. Noteworthy is a considerable increase in the value of measures, primarily sensitivity, for the music video class, 13 percentage points compared to SVM-RBF, and 21 percentage points compared to *k*-NN. All values from Table 26 were found to be statistically significant after conducting the chi-square test. 

Similarly, for the same NN configuration and the welch32 feature extraction scheme, 10-fold cross-validation was performed, and the resulted metrics are shown in Table 27.

The accuracy increased in the cross-validation-based study. The difference between original performance from the 1st scheme assessment and the lower boundary of the confidence interval for cross-validation based study is 0.0318. For the performance, drops were observed for the logic game, it was dropped by 0.027, and for the music video by 0.0014. For recall, an increase of 0.011 was observed for meditation, and the fall for the music video (i.e., 0.014). For the F1 measure, performance for meditation increased by 0.0041 and dropped by 0.0068 for music video.

#### Summary

Translation of performances from evaluation based on three subsets to assessment based on cross-validation differed in the case of all seven algorithms. Some of the changes were statistically significant, but the difference between the boundary of confidence interval and value of measure calculated based on the 1st scheme differed by a very modest amount (smaller than 0.01). Some changes were very pronounced, an example is the dwt stat-based scenario from Experiment 3. There were feature/classification algorithm scenarios which performed identically in terms of the proposed analysis, and an example of such is the one based on dwt from Experiment 2. This can be a vital indication related to how each feature extraction/algorithm can generalize while tested on data from other datasets and how reliable and reproducible these effects are.

It should be noted that applying the techniques listed below did not improve or even worsened the classification accuracy:

- adding more hidden layers,
- using parametric ReLU activation function,
- using adaptive optimization methods like Adam,
- adding batch normalization or dropout layers,
- adding L^1^ or L^2^ weight decay,
- adding additional features: skewness, kurtosis, and energy computed for every channel from raw, unprocessed frames

**Figure 2:**
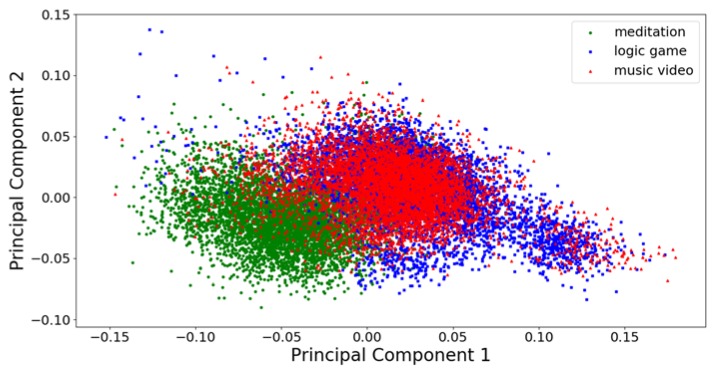
First (x-axis) and second (y-axis) principal component of the training dataset parametrized with the welch32 scheme.

**Figure 3:**
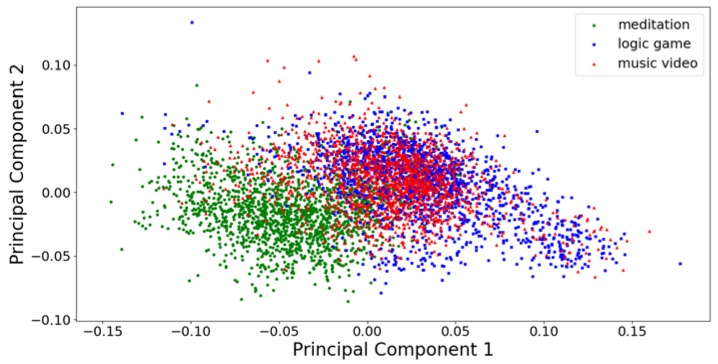
First (x-axis) and second (y-axis) principal component of the test dataset parametrized with the welch32 scheme.

In Figure 2 and Figure 3, the first two principal components of the training and test data sets parameterized with the welch32 scheme are plotted. The first two principal components, in this case, are responsible for 12.87% and 4.33% of the training dataset variance, respectively. It is possible to draw the decision boundary in such a way that most observations belonging to the classes meditation and logic game are correctly classified. Observations belonging to the music video class are problematic because they mix with observations of the other classes, in particular with the observations of the logic game class. In order to further improve the accuracy of classification, the critical issue is finding features that will enable separating observation of the music video class from the observation of the other two classes.

## 4. Conclusions

The aim of this study was to compare the effectiveness of selected methods of signal analysis and classification methods in the task of recognizing three mental states: meditation, logic game, and a video clip, based on a recorded EEG signal. The data have been preprocessed by employing independent component analysis. For parametrization of the signal, autoregressive modeling, Welch method, and discrete waveform transformation were used. Feature vectors were reduced by the principal components analysis. The classification was performed employing nearest neighbors, support vector machines, and neural networks (with three hidden layers and the LeakyReLU activation function).

Among the tested methods of signal analysis in the carried out investigation, the best results were achieved with Welch’s method, while the neural network turned out to be the most effective classifier. The choice of parameterization method turned out to have a much greater influence on the final accuracy of classification than the choice of a classifier. The same trend in metrics was also obtained while utilizing the 10-fold cross-validation scheme. We can see that for overall accuracy satisfactory results appear in our study for the meditation phase as they reach 90% in the accuracy score. This means that several limitations in our approach should be overcome; some of them are listed below.

In the conducted experiments, autoregressive model coefficients were used as features. Another possible approach is to calculate an estimate of the spectral power density from the obtained autoregressive model. Other factors that have not been studied are the effect of the ICA algorithm on classification results used [18,19,20,21,22,23,24,25,26], the effect of the initial removal of the constant component and whitening of data frames, the effect of a long data frame (also in the context of the compromise between the frame length and the number of training observations), the effect of the tab length, and in the case of the discrete waveform, the effect of the waveform. It should also be noted that another dataset should be tested as a benchmark to avoid the problem that the results obtained are due to a combination of specific features or classification techniques [98,99,100,101]. As recalled in the introductory section, there exists a variety of datasets available to the public, thus they may be utilized for this purpose, however when having similar dataset features and formats. Testing the influence of all these factors is to be a further direction of our research.

The main factor limiting the accuracy of classification was the difficulty of separating the video class observations from those of other classes. Therefore, the need to develop a set of features allowing for better separation of classes should be researched. There is also a possibility of introducing an additional meditation phase between the music video phase and the logic game. This would probably allow for a better signal separation of these two active phases, and in consequence, in a more effective classification. Moreover, analyzing all the results, one may suppose that playing the logic game and watching the music video clip result in similar brain activity. If this is a case, two classes could be discerned, i.e., meditation/activity only. This is one of the future directions of this study. 

Moreover, to determine differences, another type of BCI casque may be utilized containing more measuring electrodes and better preprocessing [44]. Then, the problem of eventually overlapping brain signals in these two activities may be easier to resolve.

Besides, it was found that EEG signals respond differently to different types of music [102]. Thus, it will be interesting to pursue this direction. This effect may also be person- and mood-dependent. That is why a questionnaire form may be prepared to ask what are subjects’ music preferences and in what mood they are when taking part in the tests. 

However, when approaching the problem of the limitations of the EEG signal analysis, and building an effective BCI interface in general, one may refer to several additional experimental issues. Zhang referred to overfitting in electroencephalogram (EEG) classification as one of the essential limitations in using EEG as brain–computer interfaces (BCIs) [35]. This may require various regularization schemes, data augmentation, or using dropouts in the NN model. Moreover, the effectiveness of the classification process depends to a large extent on the amount and quality of the prepared data (including both selection of characteristics and redundancy), thus a variety of methods might be checked with different settings. Classification outcomes determine the best configuration of the feature scheme/classification algorithm. However, for the EEG signal analysis, 2D spectral representations may be used to augment data for the deep learning classification. Another way of data augmentation is to utilize examples from similar but not identical datasets. This may allow obtaining better generalization due to exposing the network to more training examples. It may be realized based on unsupervised pre-training or transfer learning. As pointed out by Han et al. [103], it is often reasonable to assume that the input–output mapping is similar across different models, so a better NN performance may be obtained by fitting all the parameters at the same time. Lastly, since poor generalization ability still limits the broader use of BCI, thus deep learning could be employed in the form of, e.g., autoencoders without manual feature selection [32,35]. 

## Figures and Tables

**Table 1:** Examples of classification performance obtained for various tasks based on selected literature sources.

EEG-Related Task	Literature Source	Algorithm	Dataset	Classification Effectiveness
event-related potential	[46]	SVM, SWLDA, BLDA, SBL, SBLaplace	two experimental datasets	the best approach—approximately up to 100%
fatigue	[7]	spatial-temporal convolutional neural network (ESTCNN)	experimental, local dataset	97.3%
stress	[47]	DNN and deep CNN	experimental, local dataset	86.62
emotion	[48]	CNN	DEAP [49]	99.72%
emotion	[50]	dynamical graph CNN (DGCNN)	SEED [51]	90.4%
emotion	[52]	RNN with LSTM (Recurrent Neural Networks/Long Short-Term Memory	SSVEP (steady-state visually evoked potentials)	93.0%
temporal analysis	[50]	dynamical graph CNN (DGCNN)	DREAMER [53]	86.23%
sleep disturbance detection	[54]	CNN (no feature extraction)	[54]	93.55% to 98.10% depending on the number of classess
auditory stimulus classification	[55]	RNN	experimental, local dataset	83.2%
automated visual object categorization	[56]	RNN, CNN-based regressor	experimental, local dataset	83%
MI (Motor Imaginery) EEG	[57]	CNN, transfer learning	[57]	two classes: 86.49%, three classes: 79.25%, four classes: 68.51%
epileptic seizure detection	[58]	Gated Recurrent Unit RNN	BUD [58]	98%
epileptic seizure detection	[59]	Neuro-fuzzy	Local (EEG database—Bonn University) [59]	~90%
epileptic seizure detection	[60]	CNNs/LSTM	TUH EEG Seizure Corpus [61]/Duke University Seizure Corpus	sensitivity: 0.3083; specificity: 0.9686
Behavioral Disorder (RBD)	[62]	Echo State Networks (ESNs)	experimental, local dataset (118 subjects)	85%
Alzheimer disease detection	[63]	multiple convolutional-subsampling	experimental, local dataset	80%
depression screening	[64]	CNN	experimental, local dataset (patients with Mild Cognitive Impairment and healthy control group)	left hemisphere: 93.5% right hemisphere: 96%
autism	[65]	bispectrum transform, ST Fourier Transform (STFT)/STFT at a bandwidth of total spectrum (STFT-BW)	experimental, local dataset (10 autism patients and 7 control subjects)	82.4%

**Table 2:** Accuracy of test data classification with *k*-NN classifiers for chosen values of *k*.

	Feature Extraction Scheme							
*k*	ar16	ar24	dwt	dwt stat	welch16	welch32	welch64	**mean**
5	0.4742	0.4605	0.3529	0.4192	0.5999	0.6304	0.6052	0.5060
7	0.4891	0.4756	0.3559	0.4327	0.6084	0.6338	0.6145	0.5010
11	0.4941	0.4875	0.3535	0.4403	0.6163	**0.6386**	0.6245	0.5157
14	0.5030	0.4927	0.3533	0.4456	**0.6141**	0.6370	**0.6358**	0.5259
17	**0.5066**	**0.4998**	**0.3563**	**0.4569**	0.6129	0.6362	0.6322	**0.5287**
**mean**	0.4934	0.4832	0.3544	0.4389	0.6103	**0.6352**	0.6224	

**Table 3:** Results of the mixed linear model analysis for data from Table 2. The values presented are coefficients of a linear model calculated by the analysis procedure, standard error, statistic, and *p*-value of a test for statistical significance and left and right boundaries of the confidence interval for the influence of each algorithm in comparison to reference algorithm (welch32). Boundary probabilities of the confidence interval are 0.025 and 0.975.

	Coeff.	Std. Err.	z	P > |z|	Left c.f. Boundary	Right c.f. Boundary
Intercept (welch32-based influence)	0.635	0.012	53.682	0.000	0.612	0.658
ar16	−0.142	0.017	−8.474	0.000	−0.175	−0.109
ar24	−0.152	0.017	−9.082	0.000	−0.185	−0.119
dwt	−0.281	0.017	−16.782	0.000	−0.314	−0.248
dwt_stat	−0.196	0.017	−11.728	0.000	−0.229	−0.163
welch16	−0.025	0.017	−1.487	0.137	−0.058	0.008
welch64	−0.013	0.017	−0.763	0.446	−0.046	0.020

**Table 4:** Normalized confusion matrix for the 11-NN classifier and the welch32 feature extraction scheme (left). Normalized confusion matrix for the 17-NN classifier and the ar16 feature extraction scheme (right).

Confusion Matrix for 11-NN welch32					Confusion Matrix for 17-NN ar16			
	Meditation	Music Video	Logic Game			Meditation	Music Video	Logic Game
meditation	0.82	0.09	0.08		meditation	0.72	0.19	0.10
music video	0.11	0.47	0.42		music video	0.43	0.32	0.25
logic game	0.04	0.34	0.62		logic game	0.27	0.25	0.48

**Table 5:** Normalized confusion matrix for the 17-NN classifier and the dwt feature extraction scheme (left). Normalized confusion matrix for the 17-NN classifier and the dwt_stat feature extraction scheme (right).

Confusion Matrix for 17-NN dwt					Confusion Matrix for 17-NN dwt_stat			
	Meditation	Music Video	Logic Game			Meditation	Music Video	Logic Game
meditation	0.09	0.26	0.64		meditation	0.73	0.14	0.13
music video	0.08	0.26	0.67		music video	0.43	0.29	0.28
logic game	0.05	0.23	0.72		logic game	0.38	0.28	0.35

**Table 6:** Values of precision, recall, and F1 score for each signal class for chosen variants of Experiment 1.

Scenario	Class	Precision	Recall	F1
11-NN welch32	meditation	0.8497	0.8234	0.8363
	logic game	0.5521	0.6215	0.5847
	music video	0.5204	0.4710	0.4944
17-NN ar16	meditation	0.5049	0.7177	0.5928
	logic game	0.5824	0.4807	0.5267
	music video	0.4270	0.3216	0.3669
17-NN dwt	meditation	0.4274	0.0925	0.1521
	logic game	0.3545	0.7201	0.4751
	music video	0.3408	0.2563	0.2926
17-NN dwt stat	meditation	0.4772	0.7334	0.5782
	logic game	0.4593	0.3476	0.3957
	music video	0.4101	0.2896	0.3395

**Table 7:** Values of precision, recall, and F1 score in 10-fold cross-validation for the best and the worst feature extraction method variants of Experiment 1 (*k*-NN classifier).

Scenario	Class	Precision	Recall	F1
11-NN welch32	meditation	0.8621	0.8300	0.8458
	logic game	0.5794	0.5621	0.5706
	music video	0.5018	0.5354	0.5180
17-NN dwt	meditation	0.4402	0.0957	0.1572
	logic game	0.3587	0.6098	0.4517
	music video	0.3370	0.3649	0.3504

**Table 8:** Accuracy of test data classification with support vector machine (SVM)-linear classifier for chosen values of C parameter.

	Feature Extraction Scheme							
*C*	*ar16*	*ar24*	*dwt*	*dwt stat*	*welch16*	*welch32*	*welch64*	***mean***
0.01	0.5072	**0.5397**	**0.3353**	**0.5149**	0.5653	0.6122	0.6290	0.5291
0.1	0.5083	**0.5397**	0.3351	0.5129	0.6070	0.6378	0.6528	0.5491
1	**0.5085**	0.5393	0.3287	0.5131	0.6249	**0.6671**	**0.6612**	0.5490
10	**0.5085**	0.5395	-	0.5145	**0.6550**	0.6628	0.6598	0.5900
100	**0.5085**	**0.5397**	-	-	0.6548	0.6638	0.6548	**0.6043**
***mean***	0.5082	0.5396	0.3330	0.5138	0.6214	0.6487	**0.6515**	

**Table 9:** Coefficients of a linear model calculated by the analysis procedure, standard error, statistic, and *p*-value of a test for statistical significance as well as left and right boundaries of the confidence interval for the influence of each algorithm in comparison to the reference algorithm (welch64). Boundary probabilities of the confidence interval are 0.025 and 0.975.

	Coeff.	Std. Err.	z	P > |z|	Left c.f. Boundary	Right c.f. Boundary
Intercept (welch64-based influence)	0.652	0.012	53.764	0.000	0.628	0.675
ar16	−0.143	0.017	−8.291	0.000	−0.177	−0.109
ar24	−0.112	0.019	−5.965	0.000	−0.149	−0.075
dwt	−0.318	0.022	−14.386	0.000	−0.362	−0.275
dwt_stat	−0.138	0.018	−7.680	0.000	−0.173	−0.103
welch16	−0.030	0.026	−1.144	0.253	−0.082	0.021
welch32	−0.003	0.024	−0.118	0.906	−0.049	0.043

**Table 10:** Accuracy values for the case of SVM (linear kernel) in 10-fold cross-validation.

Feature Extraction Scheme		
C	dwt	welch32
0.01	0.3330	-
1	-	0.6595

**Table 11:** Normalized confusion matrix for SVM classifier with linear kernel, value of C = 1, and welch32 feature extraction scheme (left). Normalized confusion matrix for SVM classifier with linear kernel, value of C = 1, and ar16 feature extraction scheme (right).

	Meditation	Music Video	Logic Game			Meditation	Music Video	Logic Game
meditation	0.85	0.12	0.03		meditation	0.73	0.15	0.12
music video	0.13	0.50	0.37		music video	0.40	0.28	0.31
logic game	0.04	0.31	0.66		logic game	0.27	0.22	0.51

**Table 12:** Normalized confusion matrix for SVM classifier with linear kernel, value of C = 0.01, and dwt feature extraction scheme (left). Normalized confusion matrix for SVM classifier with linear kernel, value of C = 0.01, and dwt_stat feature extraction scheme (right).

	Meditation	Music Video	Logic Game			Meditation	Music Video	Logic Game
meditation	0.30	0.35	0.35		meditation	0.68	0.20	0.13
music video	0.30	0.35	0.35		music video	0.25	0.37	0.38
logic game	0.31	0.34	0.35		logic game	0.18	0.32	0.50

**Table 13:** Values of precision, recall, and F1 score for each signal class for chosen variants of Experiment 2.

Variant	Class	Precision	Recall	F1
welch32 C = 1	meditation	0.8369	0.8501	0.8434
	logic game	0.6179	0.6560	0.6364
	music video	0.5367	0.4952	0.5151
ar16 C = 1	meditation	0.5209	0.7310	0.6083
	logic game	0.5400	0.5103	0.5247
	music video	0.4360	0.2842	0.3441
dwt C = 0.01	meditation	0.3324	0.3017	0.3163
	logic game	0.3345	0.3525	0.3432
	music video	0.3388	0.3519	0.3452
dwt stat C = 0.01	meditation	0.6134	0.6753	0.6429
	logic game	0.4946	0.5000	0.4973
	music video	0.4159	0.3694	0.3913

**Table 14:** Values of precision, recall, and F1 score for 10-fold cross-validation for the best and worst results resulted from the training/validation/test scheme as contained in Table 13.

Variant	Class	Precision	Recall	F1
welch32 C = 1	meditation	0.8472	0.8594	0.8533
	logic game	0.6052	0.6246	0.6147
	music video	0.5187	0.4946	0.5063
dwt C = 0.01	meditation	0.3288	0.3134	0.3209
	logic game	0.3344	0.3394	0.3369
	music video	0.3356	0.3463	0.3408

**Table 15:** Accuracy of test data classification with the SVM-RBF classifier for chosen values of C and γ parameters.

	Feature Extraction Scheme								
*C*	*γ*	*ar16*	*ar24*	*dwt*	*dwt stat*	*welch16*	*welch32*	*welch64*	***mean***
0.01	0.1	0.3769	0.3392	0.4097	**0.3976**	0.5651	0.6133	0.6231	0.4750
	1	0.3333	0.3333	0.3414	0.3523	0.5689	0.6193	0.6332	0.4545
	10	0.4252	0.4180	0.3557	0.3333	0.6072	0.6378	0.6380	0.4879
0.1	0.1	0.4821	0.3734	0.4097	0.3976	0.5651	0.6169	0.6310	0.4965
	1	0.3333	0.3333	0.3392	0.3529	0.6161	0.6453	0.6578	0.4683
	10	0.4252	0.4178	0.3557	0.3333	0.6334	0.6683	0.6713	0.5007
1	0.1	**0.5286**	**0.5107**	0.4222	0.3333	0.6157	0.6455	0.6604	0.5309
	1	0.3597	0.3333	**0.4319**	0.3535	0.6338	0.6655	0.6709	0.4927
	10	0.3333	0.4178	0.3557	0.3333	0.6578	0.6846	**0.6866**	0.4956
10	0.1	0.4998	0.5070	-	0.3535	0.6334	0.6650	0.6626	0.5535
	1	0.3636	0.3366	-	0.3333	0.6578	0.6681	0.6765	0.5060
	10	0.3333	0.4210	-	-	0.6632	**0.6933**	0.6644	**0.5550**
100	0.1	0.5000	-	-	0.3327	0.6133	0.6632	0.6626	0.5544
	1	0.3636	-	-	0.3535	0.6548	0.6820	0.6725	0.5453
	10	0.3333	-	-	0.3333	**0.6683**	0.6701	0.6606	0.5331
**mean**		0.3994	0.3951	0.3801	0.3495	0.6236	0.6559	**0.6581**	

**Table 16:** Coefficients of a linear model calculated by the analysis procedure, standard error, statistic, and *p*-value of a test for statistical significance and left and right boundaries of the confidence interval for the influence of each algorithm in comparison to the reference algorithm (welch64). Boundary probabilities of the confidence interval (c.f. ) are 0.025 and 0.975.

	Coeff.	Std. Err.	z	P > |z|	Left c.f. Boundary	Right c.f. Boundary
Intercept (welch6-based influence)	0.658	0.042	15.639	0.000	0.576	0.741
ar16	−0.259	0.051	−5.049	0.000	−0.359	−0.158
ar24	−0.263	0.053	−4.977	0.000	−0.367	−0.159
dwt	−0.278	0.062	−4.480	0.000	−0.400	−0.156
dwt_stat	−0.309	0.062	−4.946	0.000	−0.431	−0.186
welch16	−0.035	0.064	−0.542	0.588	−0.159	0.090
welch32	−0.002	0.063	−0.035	0.972	−0.125	0.121

**Table 17:** Accuracy of test data classification for the SVM-RBF classifier for 10-fold cross-validation performed for the best and worst results obtained from the training/validation/test scheme.

Feature Extraction Scheme			
C	γ	dwt_stat	welch32
0.01	0.1	0.3229	-
10	10	-	0.6905

**Table 18:** Normalized confusion matrix for SVM classifier with RBF kernel, C = 10, γ = 10, and the welch32 feature extraction scheme (left). Normalized confusion matrix for SVM classifier with RBF kernel, C = 1, γ = 0.1, and the ar16 feature extraction scheme (right).

	Meditation	Music Video	Logic Game			Meditation	Music Video	Logic Game
meditation	0.86	0.11	0.03		meditation	0.62	0.24	0.14
music video	0.10	0.53	0.37		music video	0.28	0.37	0.35
logic game	0.01	0.30	0.69		logic game	0.18	0.22	0.59

**Table 19:** Normalized confusion matrix for SVM classifier with RBF kernel, C = 1, γ = 1, and the dwt feature extraction scheme (left). Normalized confusion matrix for SVM classifier with RBF kernel, C = 0.01, γ = 0.1, and the dwt_stat feature extraction scheme (right).

	Meditation	Music Video	Logic Game			Meditation	Music Video	Logic Game
meditation	0.70	0.16	0.14		meditation	0.52	0.26	0.22
music video	0.51	0.23	0.26		music video	0.37	0.34	0.29
logic game	0.40	0.23	0.37		logic game	0.35	0.32	0.33

**Table 20:** Values of precision, recall, and F1 score for each signal class for chosen variants of Experiment 3.

Variant	Class	Precision	Recall	F1
welch32	meditation	0.8876	0.8597	0.8735
C = 10	logic game	0.6287	0.6898	0.6578
γ = 10	music video	0.5676	0.5302	0.5483
ar16	meditation	0.5716	0.6203	0.5950
C = 1	logic game	0.5496	0.5931	0.5705
γ = 0.1	music video	0.4457	0.3724	0.4058
dwt	meditation	0.4344	0.6983	0.5356
C = 1	logic game	0.4791	0.3664	0.4152
γ = 1	music video	0.3680	0.2310	0.2838
dwt_stat	meditation	0.4178	0.5193	0.4631
C = 0.01	logic game	0.3997	0.3337	0.3638
γ = 0.1	music video	0.3685	0.3398	0.3536

**Table 21:** Values of precision, recall, and F1 score for 10-fold cross-validation for the best and worst results of the training/validation/test scheme as contained in Table 20.

Variant	Class	Precision	Recall	F1
welch32	meditation	0.8848	0.8722	0.8785
C = 10	logic game	0.6269	0.6665	0.6461
γ = 10	music video	0.5601	0.5326	0.5460
dwt_stat	meditation	0.3271	0.3925	0.3568
C = 0.01	logic game	0.3211	0.3854	0.3503
γ = 0.1	music video	0.3182	0.1909	0.2387

**Table 22:** Accuracy of test data classification using the neural network with a single hidden layer. The evaluation was repeated 10 times for each parameterization method.

ar16	ar24	dwt	dwt stat	welch16	welch32	welch64
0.5149	0.5296	0.3428	0.4911	0.6705	0.7031	0.6894
0.5191	0.5339	0.3313	0.4986	0.6721	0.7048	0.6961
0.5131	0.5266	0.3386	0.4962	0.6713	0.7046	0.6941
0.5163	0.5240	0.3400	0.4847	0.6763	0.6963	0.6913
0.5266	0.5347	0.3424	0.4974	0.6653	0.7058	0.6894
0.5208	0.5341	0.3424	0.4736	0.6755	0.7003	0.6955
0.5155	0.5236	0.3434	0.4942	0.6717	0.6997	0.6904
0.5169	0.5353	0.3428	0.4923	0.6626	0.7035	0.6870

**Table 23:** Result of the Dunn post hoc test in the form of the *p*-value matrix. Values indicating no statistically significant values are marked in bold font.

	ar16	ar24	dwt	welch16	welch32	welch64	dwt_stat
ar_16		**0.307**	0.025	0.031	<10^−3^	0.001	**0.255**
ar_24	**0.307**		0.001	**0.255**	<10^−3^	0.025	0.0301
dwt	0.025	0.001		<10^−3^	<10^−3^	<10^−3^	**0.272**
welch16	0.031	0.255	<10^−3^		0.028	**0.272**	<10^−3^
welch32	<10^−3^	<10^−3^	<10^−3^	0.028		**0.272**	<10^−3^
welch64	0.001	0.025	<10^-3^	**0.272**	**0.272**		<10^−3^
dwt_stat	**0.255**	0.031	**0.272**	<10^−3^	<10^−3^	<10^−3^	

**Table 24:** Specifications of neural networks for which the highest values of classification accuracy were achieved.

Hidden Layers	Activation Function	SGD Parameters	Patience	Max Epochs	Accuracy
3	LReLU (a = 0.2)	*lr* = 0.01 decay = 10^−6^ momentum = 0.9	50	2000	**0.7477**
4	tanh + LReLU (a = 0.2)	*lr* = 0.005 decay = 10^−6^ momentum = 0.9	250	3000	0.7469
6	ReLU	*lr* = 0.01 decay = 10^−6^ momentum = 0.9	70	2000	0.7467
3	tanh	*lr* = 0.01 decay = 10^−6^ momentum = 0.9	250	3000	0.7446

**Table 25:** Normalized confusion matrix for NN with three hidden layers, LeakyReLU activation function, and the welch32 feature extraction scheme (on the left side: training/validation/test scheme is shown, whether the outcomes of 10-fold cross-validation are contained on the right side).

Training/Validation/Test	Meditation	Music Video	Logic Game	10-Fold Cross-Validation	Meditation	Music Video	Logic Game
meditation	0.87	0.11	0.02	meditation	0.90	0.08	0.02
music video	0.06	0.69	0.26	music video	0.07	0.63	0.29
logic game	0.02	0.30	0.68	logic game	0.02	0.29	0.69

**Table 26:** Values of precision, recall, and F1 score for each class for NN with three hidden layers with the LeakyReLU activation function and the welch32 feature extraction scheme.

Class	Precision	Recall	F1 Score
meditation	0.9203	0.8724	0.8957
logic game	0.7116	0.6832	0.6971
music video	0.6296	0.6874	0.6572

**Table 27:** Values of precision, recall, and F1 score for each activity class for NN with three hidden layers with the LeakyReLU activation function and the welch32 feature extraction scheme (10-fold cross-validation).

Class	Precision	Recall	F1 Score
meditation	0.9079	0.8973	0.9026
logic game	0.6840	0.6933	0.6886
music video	0.6343	0.6332	0.6337

## Supplementary Materials

Python code prepared for the experiments is available at https://multimed.org/research/sensors2020.zip.

## Appendix A

Appendix A contains simplified snippets of code utilized in experiments.

The FastICA algorithm is shown below:

# independent component analysis with whitening
# performed for single frame
from sklearn.decomposition import FastICA
ica = FastICA(n_components=14, whiten=True)
new_dataframe = ica.fit_transform(dataframe)

The following code was employed concerning calculations of the autoregressive models. Only the real values of computed model coefficients were utilized. After concatenating model coefficients from all channels with previously computed mean values and variances final feature vectors of 252 elements were obtained.

# computing autoregressive models for single frame
import spectrum
new_dataframe = []
for channel in dataframe:
  model = spectrum.arburg(channel,order=16,criteria=None)[0]
  model = [item.real for item in model]
  new_dataframe.append(model)

In the case of the welch16 parametrization method, samples from each channel in every frame were divided into eight non-overlapping subframes of 16 samples each. Subsequently, nine coefficients were obtained per every channel. Final feature vectors (with pre-computed mean values and variances) contained 154 elements:

# computing power spectral density with Welch’s method for single frame
import scipy.signal
psd = scipy.signal.welch(dataframe,nperseg=16,axis=0)[1]

Decompositon into wavelets was performed using the 4th level discrete wavelet transform (*wavedec* function from *pywt* library). After concatenating coefficient vectors with pre-computed mean values and variances, final feature vectors of 2184 elements were obtained. The transcription of this algorithm is provided in a listing below:

# computing discrete wavelet transform
# for single frame
import pywt, numpy as np
new_dataframe = []
for channel in dataframe:
   dwt = pywt.wavedec(channel,wavelet=“db4”,level=4,axis=0)
   vector = np.ndarray((0,))
   for item in dwt:
      vector = np.append(vector,item)
      new_dataframe.append(vector)

In the case of dwt_stat parametrization method, for each of five wavelet coefficient-based vectors mean value, mean value of absolute values, variance, skewness, kurtosis, zero-crossing rate, and the sum of squares were computed:

# computing descriptive parameters from wavelet coefficients
# for single frame
import numpy as np, scipy.stats, pywt
def zero_crossings(data):
    return ((data[:−1] ∗ data[1:]) < 0).sum()
dwt    = pywt.wavedec(channel,wavelet=“db4”,level=4,axis=0)
vector   = []
for item in dwt:
  vector.append(np.mean(item))
  vector.append(np.mean(np.abs(item)))
  vector.append(np.var(item))
  vector.append(scipy.stats.skew(item))
  vector.append(scipy.stats.kurtosis(item))
  vector.append(zero_crossings(item))
  vector.append(sum(np.power(item,2)))

Dimensionalities of feature vectors obtained with the aforementioned schemes were reduced via principal component analysis (PCA), this was performed in Python as follows:

# dimensionality reduction via principal component analysis
from sklearn.decomposition import PCA
pca        = PCA(n_components=0.95)
pca.fit(train_data)
reduced_train_data = pca.transform(train_data)
reduced_val_data = pca.transform(val_data)
reduced_test_data   = pca.transform(test_data)

Validation tests were performed as follows:

# dividing dataset into training, validation and test data
import random
  data_train = {}, data_val = {}, data_test = {}
  a     = int(0.7 ∗ 8265)
  b     = a + int(0.1 ∗ 8265)
for class_name in eeg.keys():
# eeg.keys() are [‘meditation’,‘music_video’,‘logic_game’]
  indices = [i for i in range(8265)]
  random.shuffle(indices)
  data_train[class_name] = [eeg[class_name][i] for i in indices[:a]] data_val[class_name] = [eeg[class_name][i] for i in indices[a:b]]
  data_test[class_name] = [eeg[class_name][i] for i in indices[b:]]

In the case of training neural networks, it is required for the inputs and outputs of the network to be encoded as vectors of numbers. An example of one-hot encoding is shown below:

# encoding classes via one-hot encoding
classes = {}
classes[‘meditation’] = np.array([1,0,0])
classes[‘music_video’] = np.array([0,1,0])
classes[‘logic_game’] = np.array([0,0,1])
for i in range(len(train_data_classes)):
  train_data_classes[i] = classes[train_data_classes[i]]
for i in range(len(test_data_classes)):
  test_data_classes[i] = classes[test_data_classes[i]]
for i in range(len(val_data_classes)):
  val_data_classes[i] = classes[val_data_classes[i]]

Code for training *k*-NN classifiers, test data classification, and computing score measures in Experiment 1 is shown below:

# training *k*-NN classifiers, test data classification
# computing accuracy, confusion matrix, precision, recall and F1
from sklearn.neighbors import KNeighborsClassifier
from sklearn.metrics import accuracy_score, confusion_matrix, classification_report
knn      = KNeighborsClassifier(n_neighbors=k)
knn.fit(reduced_train_data,train_data_classes)
results    = knn.predict(reduced_test_data)
score      = accuracy_score(test_data_classes,results)
conf_matrix   = confusion_matrix(test_data_classes,results)
report    = classification_report(test_data_classes,results)

The code for training and testing SVM classifiers (Experiment 2, linear kernel function) is shown below:

# training SVM classifiers with linear kernel
# and test data classification from sklearn.svm import SVC
svm   = SVC(C=C,kernel=‘linear’)
svm.fit(reduced_train_data,train_data_classes)
results = svm.predict(reduced_test_data)

The code for training and testing SVM classifiers (Experiment 3, radial kernel function) is shown below:

# training SVM classifier with radial basis function kernel
# and test data classification from sklearn.svm import SVC
svm  = SVC(C=C,kernel=‘rbf’,gamma=gamma)
svm.fit(reduced_train_data,train_data_classes)
results = svm.predict(reduced_test_data)

The code employed for for training and testing neural networks (Experiment 4) is provided below:

# traning neural networks with single hidden layer
# and training data classification
from keras.layers import Dense, Activation from keras.models import Sequential
from keras.optimizers import SGD
from keras.callbacks import EarlyStopping n_inputs = reduced_train_data.shape[1] model = Sequential()
model.add(Dense(n_inputs,activation=‘relu’,input_dim=n_inputs,
kernel_initializer=‘he_uniform’,bias_initializer=‘zeros’))
  model.add(Dense(3,activation=‘softmax’,
kernel_initializer=‘he_uniform’,bias_initializer=‘zeros’))
  sgd = SGD(lr=0.01,decay=1e-6,momentum=0.9,nesterov=True)
model.compile(loss=‘categorical_crossentropy’, optimizer=sgd,metrics=[‘accuracy’])
es = EarlyStopping(monitor=‘val_loss’,mode=‘min’,patience=50,restore_best_weights=True)
history = model.fit(reduced_train_data,train_data_classes,batch_size=64,
  validation_data=(reduced_val_data,val_data_classes), callbacks=[es],epochs=2000)
predictions = model.predict(reduced_test_data,batch_size=128)
score   = model.evaluate(reduced_test_data, test_data_classes,batch_size=128)
